# Polymer-based restorative outcomes following deep margin elevation: a systematic review and meta-analysis

**DOI:** 10.3389/fdmed.2026.1854285

**Published:** 2026-07-02

**Authors:** Rim Bourgi, Carlos Enrique Cuevas-Suárez, Ahmed Tarek Farouk, Juan Carlos Hernández-Cabanillas, José de Jesús Navarrete-Hernández, Elena Saraía Baena-Santillán, Antony Chidiac, Celso Afonso Klein Junior, Ahmed A. Holiel

**Affiliations:** 1 Department of Restorative and Esthetic Dentistry, Faculty of Dental Medicine, Saint-Joseph University of Beirut, Beirut, Lebanon; 2 Department of Biomaterials and Bioengineering, INSERM UMR_S 1121, University of Strasbourg, Strasbourg, France; 3 Department of Restorative Sciences, Faculty of Dentistry, Beirut Arab University, Beirut, Lebanon; 4 Dental Materials Laboratory, Academic Area of Dentistry, Autonomous University of Hidalgo State, Circ ito Ex Hacienda la Concepción S/N, San Agustín Tlaxiaca, Hgo, Mexico; 5 Assistant Professor at the Restorative Dentistry Department, Misr International University, Obour city, Egypt; 6 Health Sciences Faculty, Autonomous University of Baja California, Tijuana, BC, Mexico; 7 Department of Oral Surgery and Implantology, Faculty of Dental Medicine, Saint-Joseph University, Beirut, Lebanon; 8 Postgraduate Program in Dentistry, Universidade Luterana do Brazil (ULBRA), Canoas, Rio Grande do Sul, Brazil; 9 Department of Conservative Dentistry, Faculty of Dentistry, Alexandria University, Alexandria, Egypt

**Keywords:** adhesive dentistry, cervical margin relocation, composite resin, deep margin elevation, dental polymers, indirect restorations, restoration survival

## Abstract

**Background:**

Deep margin elevation (DME), also known as cervical margin relocation or proximal box elevation, has been proposed as a minimally invasive approach for managing subgingival margins and facilitating adhesive placement of indirect restorations. By relocating deep proximal margins coronally, DME improves isolation and enhances bonding procedures. The technique relies primarily on resin-based polymeric materials, including conventional, flowable, bulk-fill, and injectable composites, as well as resin-modified glass ionomer cements. These materials exhibit distinct polymerization behavior, shrinkage stress, and interfacial properties, which may influence marginal adaptation and long-term clinical performance. However, the clinical relevance of these material-dependent differences remains unclear.

**Objective:**

This systematic review and meta-analysis aimed to evaluate the clinical performance of indirect restorations placed after DME, with particular emphasis on the role of polymer-based restorative materials.

**Methods:**

A comprehensive electronic search of PubMed/MEDLINE, Scopus, Web of Science, Scielo, and Embase was conducted from inception to March 2026. Randomized clinical trials and prospective or retrospective clinical studies assessing indirect restorations placed following DME in permanent teeth were included. Primary outcomes were restoration survival and success. Secondary outcomes included marginal adaptation, secondary caries, postoperative sensitivity, periodontal parameters, and technical complications. Risk of bias was assessed using the Cochrane Risk of Bias 2 (RoB 2) and Risk Of Bias In Non-randomized Studies—of Interventions (ROBINS-I) tools, and the certainty of evidence was evaluated using the Grading of Recommendations, Assessment, Development and Evaluation (GRADE) approach. When applicable, meta-analysis was performed using a random-effects model, and heterogeneity was assessed using Cochran's *Q* and *I*^2^ statistics.

**Results:**

Ten clinical studies involving approximately 623 teeth met the inclusion criteria. Restoration survival ranged from 93% to 100% over follow-up periods of 6 months to 12 years. Resin-based composite materials, including flowable, bulk-fill, and injectable systems, demonstrated comparable clinical performance in terms of survival, marginal integrity, and periodontal stability. No statistically significant differences were observed for secondary caries or postoperative sensitivity (*p* > 0.05). Periodontal parameters remained generally stable. Although a statistically significant increase in bleeding on probing at relocated margins was observed in the meta-analysis (*p* = 0.02), the absolute increase was clinically modest (mean BoP ranging from 12% to 24% at DME sites vs. 8% to 16% at control sites). Probing depths remained below 4 mm in all groups, with no significant attachment loss or radiographic bone resorption. The risk of bias varied between studies included in this review. GRADE assessment indicated moderate certainty of evidence for survival, marginal adaptation, periodontal outcomes, and complications, and low certainty for secondary caries and postoperative sensitivity.

**Conclusions:**

Current clinical evidence supports the use of adhesive polymer-based materials for DME, demonstrating predictable outcomes in the restoration of teeth with deep proximal margins. Despite differences in polymer composition and polymerization characteristics, no clear superiority among materials was identified. Nevertheless, the limited number of randomized trials and methodological heterogeneity highlight the need for well-designed long-term studies to better understand the influence of polymer-related properties on clinical outcomes.

**Systematic Review Registration:**

identifier CRD420261350880.

## 1 Introduction

The management of deep proximal carious lesions and subgingival cavity margins remains a persistent clinical challenge in restorative dentistry. When restorative margins extend apically beyond the cemento-enamel junction, adequate isolation, adhesive bonding, and impression accuracy become compromised. This clinical scenario is further complicated by limited visibility, contamination by crevicular fluid, and difficulties in achieving predictable marginal sealing, all of which directly influence the longevity of indirect restorations (1, 2). Historically, surgical crown lengthening and orthodontic extrusion were recommended to reposition subgingival margins coronally and facilitate restorative procedures. However, these approaches are invasive, may compromise periodontal support and esthetics, alter crown-root ratios, and negatively affect esthetics, particularly in posterior teeth (3).

Deep margin elevation (DME), also referred to as cervical margin relocation (CMR) or proximal box elevation (PBE), was introduced as a conservative alternative to surgical intervention. The technique involves the coronal relocation of deep cervical margins using an adhesive restorative material prior to the placement of an indirect restoration (4). While DME is most commonly described in the literature as an adjunct to indirect restorations (e.g., ceramic or composite onlays, inlays, or crowns), it is also used clinically in combination with direct composite restorations, particularly for deep proximal Class II cavities where isolation and access for incremental composite placement are otherwise challenging (2, 8). In such cases, the elevated margin facilitates proper contouring, finishing, and polishing of the direct restoration. The present systematic review focuses on DME performed prior to indirect restorations, as this represents the majority of the available clinical evidence.

By relocating a deep cervical margin coronally to an equigingival or supragingival position, DME improves access for adhesive procedures, enhances marginal sealing, and facilitates impression or digital scanning procedures required for indirect restorations while preserving periodontal architecture (5, 6). In contemporary adhesive dentistry, DME has emerged as a minimally invasive strategy that aligns with the principles of tissue preservation and biomimetic restoration (2).

The biological and mechanical success of DME depends on several factors, including margin depth, adhesive strategy, isolation protocol, and the type of restorative material used for elevation. Improper technique or material selection may negatively affect marginal adaptation, periodontal response, and long-term restoration survival (7, 8). Resin composites are currently considered the material of choice due to their adhesive properties, favorable mechanical strength, and ability to bond predictably to dentin (9). Nonetheless, variations in viscosity, filler content, polymerization kinetics, and elastic modulus among conventional, flowable, and bulk-fill composites may influence marginal adaptation and stress distribution at the tooth-restoration interface (1). In addition, resin-modified glass ionomer cements have been proposed because of their chemical adhesion and fluoride release, although concerns persist regarding their mechanical stability beneath indirect restorations. These material-dependent differences may have critical implications for interfacial integrity, stress dissipation, and long-term clinical success, particularly in deep subgingival environments (10).

While numerous *in vitro* studies have investigated microleakage, fracture resistance, and bond strength in DME models (11–13), clinical evidence remains limited and fragmented. Importantly, it is unclear whether the selection of elevation material significantly influences long-term restoration survival, marginal integrity, secondary caries incidence, or periodontal response under clinical conditions. Consequently, robust clinical evidence is needed to guide material selection and optimize treatment outcomes. As DME gains increasing acceptance in adhesive dentistry, a critical synthesis of clinical outcomes according to elevation material becomes essential for evidence-based decision-making.

Therefore, the aim of this systematic review and meta-analysis was to evaluate the clinical performance of indirect restorations placed after DME in permanent teeth. This review specifically focuses on clinically relevant outcomes, including restoration survival, marginal integrity, and periodontal response, to provide a comprehensive evidence-based framework for this restorative technique. The null hypothesis tested was that no statistically significant differences exist between indirect restorations placed after DME and indirect restorations placed supragingival with respect to restoration survival, marginal adaptation, secondary caries incidence, postoperative sensitivity, and periodontal parameters.

## 2 Materials and methods

### 2.1 Study design

This systematic review and meta-analysis was conducted and reported in accordance with the Preferred Reporting Items for Systematic Reviews and Meta-Analyses (PRISMA) 2020 guidelines (14) and the Cochrane Handbook for Systematic Reviews of Interventions (15). The review protocol was prospectively registered in the PROSPERO database under CRD420261350880 registration number.

The research question was formulated using the Population, Intervention, Comparison, Outcomes, and Study design (PICOS) framework. The population comprised patients receiving indirect restorations in permanent teeth. The intervention consisted of DME using adhesive restorative materials, including conventional resin composites, flowable composites, bulk-fill composites, and resin-modified glass ionomer cements, followed by placement of an indirect restoration (e.g., ceramic or composite onlays, inlays, crowns, or overlays). The comparison involved indirect restorations placed supragingival. The primary outcomes included restoration survival and marginal adaptation. Secondary outcomes encompassed secondary caries, postoperative sensitivity, periodontal parameters (probing depth, bleeding on probing, attachment level), and restoration-related biological or technical complications. Eligible study designs included randomized controlled trials, controlled clinical trials, and prospective or retrospective cohort studies with clinical follow-up.

### 2.2 Search strategy

A comprehensive electronic search was performed in PubMed/MEDLINE, Scopus, Web of Science, Scielo and Embase from database inception to March 2026. The search strategy combined controlled vocabulary terms and free-text keywords related to DME and clinical performance outcomes. The detailed PubMed search strategy is presented in Table 1 and was adapted for use in the other databases.

**Table 1:** Search strategy performed at pubMed and adapted to other databases.

Search	Terms
#1 (Deep margin elevation technique)	“deep margin elevation” OR “cervical margin relocation” OR “margin relocation technique” OR “proximal box elevation”
#2 (Elevation materials)	“resin composite” OR “composite resin” OR “flowable composite” OR “bulk-fill composite” OR “resin-modified glass ionomer” OR “RMGIC”
#3 (Restorative context)	“indirect restoration” OR “ceramic restoration” OR “indirect composite restoration” OR “onlay” OR “inlay” OR “overlay”
#4 (Clinical outcomes)	“clinical performance” OR “clinical evaluation” OR “restoration survival” OR “marginal adaptation” OR “secondary caries” OR “periodontal response” OR “postoperative sensitivity”
#5 (Combined)	#1 AND #2 AND #3 AND #4

### 2.3 Study selection

Two independent reviewers (R.B. and A.A.H.) independently screened titles and abstracts of all retrieved records. Full-text articles of potentially eligible studies were then assessed according to predefined inclusion and exclusion criteria. Studies were included if they were clinical investigations evaluating indirect restorations (e.g., ceramic or composite onlays, inlays, crowns, or overlays) placed following DME in permanent teeth, and reporting at least one relevant clinical outcome with a minimum follow-up of six months. Exclusion criteria included *in vitro* studies, animal studies, case reports, case series without comparison groups, narrative or systematic reviews, and studies evaluating surgical margin relocation rather than restorative DME. Disagreements between reviewers were resolved through discussion or consultation with a third reviewer (C.E.C.-S.) until consensus was reached.

### 2.4 Data extraction

Data were independently extracted by two reviewers (A.A.H., R.B.) using a standardized data collection form. Extracted data included study characteristics (first author, year, country), study design (Randomized controlled trial, prospective, retrospective), sample size (number of patients/teeth), type of elevation material and manufacturer, adhesive strategy employed, type of indirect restoration, margin location (supragingival, equigingival, subgingival depth), follow-up duration, and reported clinical outcomes (survival/failure, marginal adaptation, secondary caries, periodontal parameters, postoperative sensitivity, complications). When necessary, authors were contacted for clarification or additional information. Extracted data were summarized in structured tables to ensure transparency and reproducibility.

### 2.5 Risk of bias assessment

Methodological quality assessment and risk of bias were independently performed for each included study by a blinded examiner using established critical appraisal tools. The quality of randomized clinical trials was assessed using the Cochrane Collaboration's Risk of Bias 2 (RoB 2) tool. Consensus on scoring was achieved through discussion among all reviewers prior to the assessments (16). For non-randomized prospective and retrospective clinical studies, the Risk Of Bias In Non-randomized Studies—of Interventions (ROBINS-I) tool was applied to assess the risk of bias. This tool evaluates bias across seven domains, including bias due to confounding, selection of participants, classification of interventions, deviations from intended interventions, missing data, measurement of outcomes, and selection of the reported result (17). Each domain was assessed to determine the overall risk of bias for the included non-randomized studies.

### 2.6 Certainty of evidence assessment

The certainty of evidence for each clinical outcome was evaluated using the Grading of Recommendations, Assessment, Development and Evaluation (GRADE) approach (18). Factors considered included study limitations, inconsistency, indirectness, imprecision, and potential publication bias. The quality of evidence was classified as high, moderate, low, or very low, and detailed justifications for downgrading were reported.

### 2.7 Data synthesis

The meta-analyses were performed using Review Manager Software version 5.1 (The Nordic Cochrane Centre, The Cochrane Collaboration, Copenhagen, Denmark). A random-effects model was applied for all analyses to account for potential clinical and methodological heterogeneity among the included studies. Pooled-effect estimates were calculated using the risk difference (RD) of failure of indirect restorations performed after deep margin elevation (DME) compared with the control group. For this purpose, data from each study were dichotomized as acceptable or unacceptable. The number of unacceptable outcomes (events) and the total number of restorations per group were used to compute the risk difference. Separate meta-analyses were conducted for specific categories of restoration evaluation, including fracture or debonding, secondary caries, and gingival inflammation. Subgroup analyses were performed according to follow-up duration. Statistical significance was set at *p* ≤ 0.05. Statistical heterogeneity among studies was assessed using Cochran's *Q* test and the inconsistency (*I*^2^) statistic.

## 3 Results

### 3.1 Study selection

**Figure 1:**
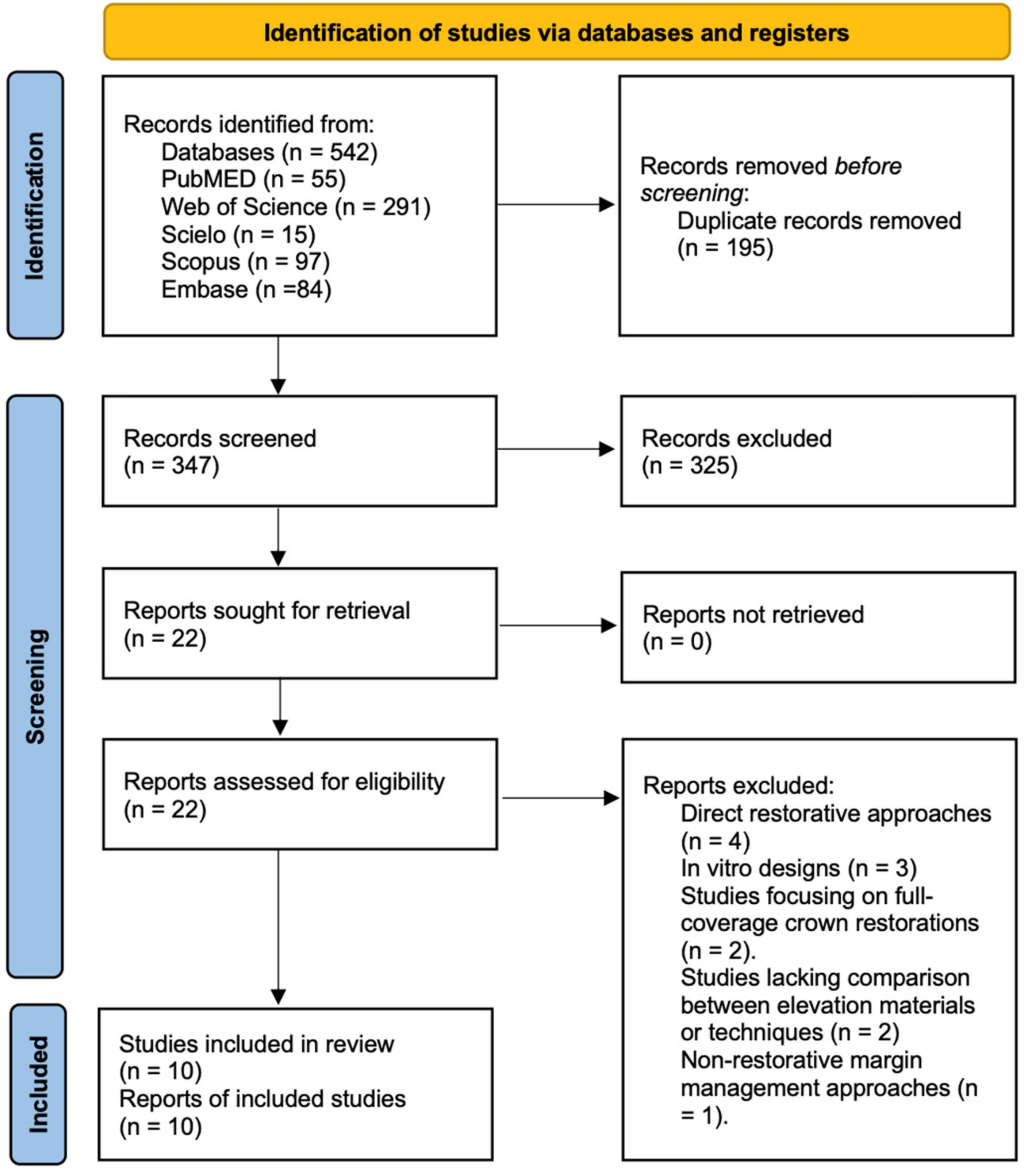
PRISMA 2020 flow diagram of study selection.

The systematic search yielded 542 records from PubMed/MEDLINE, Scopus, Web of Science, EMBASE and Scielo. After removal of 195 duplicates, 347 unique records were identified. Following title and abstract screening, 325 records were excluded. Subsequently, 22 full-text articles were assessed for eligibility according to the predefined inclusion and exclusion criteria. After full-text review, 11 studies were excluded. The main reasons for exclusion at this stage included *in vitro* studies, absence of DME procedures, evaluation of non-restorative margin management approaches (e.g., surgical crown lengthening), and absence of relevant clinical outcomes or follow-up data. Ultimately, 10 clinical studies (including randomized controlled trials, prospective, and retrospective clinical studies) evaluating the clinical performance of restorations placed following DME were included in the qualitative and quantitative synthesis (5, 6, 10, 19–25). The study selection process is summarized in the PRISMA flow diagram (Figure 1), detailing the number of records identified, screened, excluded, and included in the final analysis.

### 3.2 Study characteristics

The included clinical studies comprised randomized controlled trials, prospective controlled clinical trials, prospective clinical studies, and retrospective analyses evaluating DME in posterior teeth restored with indirect restorations. Follow-up periods ranged from 6 months to 12 years, and most studies assessed restorations placed in molars and premolars with deep proximal subgingival margins (5, 6, 10, 19–25). A summary of the key study characteristics is presented in Table 2. Information includes study design, elevation material, adhesive strategy, type of indirect restoration, comparator groups, sample size, follow-up duration, and reported clinical and periodontal outcomes. The majority of studies investigated composite-based DME techniques using flowable, bulk-fill, or injectable materials in combination with contemporary adhesive systems and resin-based luting protocols.

**Table 2:** Characteristics of the studies included in the review.

Study (Author, Year, Country)	Study design	Sample size (patients/teeth)	Elevation material (manufacturer, country)	Adhesive strategy	Indirect restoration type	Margin location	Follow-up	Clinical outcomes reported	Key findings
Ferrari et al., 2018, Italy (19)	Prospective controlled clinical trial	35/35 posterior teeth	Universal flow resin composite (GC Co., Tokyo, Japan)	Universal adhesive (G-Premio Bond, GC) + resin cement (LinkForce, GC)	Pressed lithium disilicate partial crowns (LiSi Press, GC Co., Tokyo, Japan)	DME vs. shoulder preparation (below CEJ)	Baseline, 12 months	Bleeding on probing (BoP) Gingival Index (GI) Plaque Index (PI) Periodontal probing depth (PPD) Margin–bone crest distance Radiographic findings	DME group showed higher BoP at 12 months. GI and PI were similar between groups. PPD increased slightly in both groups. Most positive BoP sites in Group 1 had margin-bone crest distance of 2 mm. No secondary caries or radiographic anomalies detected. DME is associated with increased BoP, especially when margins are close to bone crest, indicating clinically sensitive technique for DME
Bresser RA, 2019, Netherlands (20)	Retrospective clinical study	120/197 posterior teeth	Tetric EvoCeram (Ivoclar Vivadent); Estelite *Σ* Quick (Tokuyama Dental, Tokyo, Japan)	Three-step etch-and-rinse (OptiBond FL, Kerr) + immediate dentin sealing + adhesive luting with preheated composite	Lithium disilicate (IPS e.max) and indirect composite (Adoro) partial restorations	Subgingival margins relocated supragingivally (DME)	Up to 12 years (mean 57.7 months)	Survival, restoration adaptation, USPHS criteria (adaptation, color match, margin discoloration, fracture of restoration/tooth, wear, antagonist wear, secondary caries, postoperative sensitivity, periodontal health)	Overall survival 95.9% up to 12 years. Eight failures (secondary caries, fracture, pulpal necrosis, periodontal breakdown). Ceramic restorations showed less degradation but more antagonist wear. DME enabled predictable bonding and isolation with long-term performance comparable to conventional margins.
Farouk AT, 2023, Egypt (21)	RCT	20/20 proximal cavities	SDR® Flow + (Dentsply Sirona, Germany) + 3 M™ Filtek™ One Bulk Fill Restorative (3 M ESPE, USA)	Single Bond Universal adhesive (3 M ESPE, USA), selective etch mode; adhesive cementation with injectable composite (Essentia LoFlo, GC Corp., Japan)	CAD/CAM composite overlays (Brilliant Crios 14 HT, Coltene, Switzerland)	Subgingival margin elevated with DME vs. surgical crown lengthening (CL)	Baseline, 1, 3, 6, 9, 12 months	Clinical attachment level (CAL), probing depth (PD), bleeding on probing (BOP), crestal bone level (CBL), secondary caries, periodontal response	DME group demonstrated stable and lower CAL values compared to CL after 3 months through 12 months. PD decreased significantly over time in DME, showing lower values than CL at 9–12 months. BOP prevalence decreased progressively in both groups, reaching 0% at 12 months. CBL changes were minimal and slightly lower in DME than CL, reflecting high bone stability. No secondary caries were reported. DME provided a conservative, biologically favorable alternative to CL, maintaining periodontal attachment with minimal tissue disruption.
Gözetici-Çil B, 2024, Turkey (22)	Prospective clinical trial	63/80 posterior teeth	Tetric-N Flow + Tetric-N Ceram (Ivoclar Vivadent, Schaan, Liechtenstein)	Three-step adhesive (Syntac Primer/Adhesive/Heliobond, Ivoclar Vivadent) + dual-polymerizing resin cement (Variolink-N, Ivoclar Vivadent)	Laboratory-processed partial indirect resin composite restorations (SR Nexco, Ivoclar Vivadent)	Deep proximal subgingival margins elevated coronally using DME.	Baseline, 6 months, 1, 2, 3 years	Restoration survival, Marginal adaptation, marginal discoloration, secondary caries, surface luster, occlusal wear, anatomical form, fracture/retention, postoperative sensitivity, tooth integrity	Overall survival 93.7%, success 87.5%. Ten failures observed: 9 fractures, 1 secondary caries. Minor marginal adaptation irregularities and slight marginal discoloration increased over time. No significant difference in survival related to endodontic status or periodontal grade. Higher failure risk with overlays (HR = 8.55, *p* = 0.023) and one-sided DME (HR = 4.90, *p* = 0.047).
Hausdörfer T, 2024, Germany (5)	Prospective controlled clinical trial	68/77 posterior teeth	Flowable bulk-fill composite DME (SDRflow; Dentsply Sirona, Germany)	3-step etch & rinse adhesive (Optibond FL; Kerr, Switzerland) + universal primer and dual-cure luting composite (Monobond Plus + Variolink esthetic DC; Ivoclar Vivadent, Liechtenstein)	CAD/CAM partial lithium disilicate restorations (IPS e.max CAD; Ivoclar Vivadent, Liechtenstein)	Supragingival/Equigingival control vs. subgingival relocated with DME	Baseline, 1-year	Bleeding on probing (BoP), periodontal probing depth (PPD), plaque index (PI), Restoration failure (ceramic fracture, secondary caries), Loss of retention	DME sites showed significantly increased BOP at 1-year. PI increased on all surfaces but did not differ between DME and control. PPD remained stable. Two restorations failed (1 fracture, 1 secondary caries); three restorations showed temporary loss of retention and were reluted. DME demonstrated predictable clinical performance with slightly increased gingival inflammation but overall high survival and patient satisfaction at 1-year follow-up.
El-Ma'aita AM, 2024, Jordan (6)	Retrospective clinical study	25/28 teeth	Preheated Filtek Z250 (3M, St. Paul, MN, USA)	Universal adhesive (Scotchbond Universal, 3M) + dual-cure resin cement (RelyX Ultimate, 3M)	Lithium disilicate or monolithic zirconia crowns/endocrowns/overlays	Subgingival margins relocated supragingivally (DME)	12–47 months (mean 25.4 months)	Restoration adaptation, marginal discoloration, recurrent caries, gingival inflammation, plaque accumulation, probing depth, periodontal health, apical pathology, restoration success	Overall success 96.4%. All DME restorations adapted well with no recurrent caries or marginal discoloration. Four DME and four non-DME sites showed moderate gingival inflammation. Mean probing depth 2.8 mm (DME) vs. 2.6 mm (non-DME), no statistical difference. One tooth developed apical pathology (acute abscess), otherwise no failures. DME demonstrated safe short- to mid-term clinical and periodontal outcomes comparable to non-DME sites.
Aksaka N, 2025, Turkey (23)	Prospective controlled clinical trial	39/42 molars	Tetric-N Flow + Tetric-N Ceram (Ivoclar Vivadent, Schaan, Liechtenstein)	Three-step etch-and-rinse adhesive (Syntac Primer/Adhesive & Heliobond, Ivoclar Vivadent) + dual-cure resin cement (Variolink N, Ivoclar Vivadent)	Laboratory-processed indirect resin composite restorations (SR Nexco, Ivoclar Vivadent)	Subgingival elevated coronally with DME vs. natural proximal surfaces (control)	Baseline, 6, 12, 24, 36 months	Plaque Index, Gingival Index, Probing Pocket Depth (PPD), bleeding on probing, radiographic bone level	No significant differences in PI, GI, PPD, or BoP between DME and control at any time point. PPD showed time-dependent changes but no group–time interaction. No radiographic bone loss observed. BoP values remained high but comparable between groups. DME considered biologically safe and clinically acceptable, even when margins approached STA.
Hausdörfer T, 2025, Germany (24)	Prospective controlled clinical study	68/77 restorations (57 evaluated at 2 years)	SDRflow (Dentsply Sirona, Bensheim, Germany)	Etch & rinse 3-step adhesive (Optibond FL, Kerr, Bioggio, Switzerland) + Universal adhesive for luting (Adhese Universal, Ivoclar Vivadent, Schaan, Liechtenstein) + Dual-curing resin cement (Variolink DC, Ivoclar Vivadent, Schaan, Liechtenstein)	CAD/CAM partial lithium disilicate restorations (IPS e.max CAD, Ivoclar Vivadent, Schaan, Liechtenstein)	Subgingival elevated with DME vs. supragingival/equigingival control	Baseline, 1 year, 2 years	Survival, FDI clinical scores, Bleeding on Probing (BOP), Plaque Index (PI), Probing Pocket Depth (PPD), Secondary caries, Restoration fracture	Survival at 2 years: 95.2%; 2 restorations failed (1 fracture, 1 secondary caries), 1 tooth with apical periodontitis. BOP increased at DME sites (padj=0.010) but not significantly different vs. control (padj=0.110). PI increased in both groups (padj < 0.001), PD higher at DME (padj=0.015) but stable over time. Overall, DME restored teeth showed comparable periodontal stability to control with minor increases in gingival inflammation.
Adson O, 2026, Turkey (25)	Prospective clinical study	88/100 posterior teeth	G-ænial Universal Injectable (GC Corp., Tokyo, Japan)	Universal adhesive (G-Premio Bond, GC) + dual-cure self-adhesive resin cement (G-CEM ONE, GC)	CAD/CAM hybrid ceramic endocrown-like restorations (Cerasmart 270, GC)	Supragingival (SGM) vs. subgingival (SubGM) relocated with DME	6, 12, 24 months	Marginal adaptation/discoloration, surface texture, anatomical form, secondary caries, color match, gingival inflammation, patient satisfaction, survival	Overall survival 97% (SGM 98%; DME 96%). Five failures (3 debondings, 2 fractures). No secondary caries. No significant difference between groups. DME demonstrated clinical performance comparable to supragingival margins with high patient satisfaction and stable periodontal response over 24 months.
Roshdy BN, 2026, Egypt (10)	RCT (multi-arm, parallel, double-blind)	64/64 molars	Highly viscous glass ionomer Equia Fil; High-filled injectable composite G-ænial Universal Injectable; RMGI Fuji II LC (GC Corp., Tokyo, Japan); Activa Bioactive Restorative (Pulpdent Corp., Watertown, MA, USA)	Universal adhesive (G-Premio Bond, GC) with immediate dentin sealing + dual-cure self-adhesive resin cement (Bifix SE, VOCO, Germany); selective enamel etching	CAD/CAM nanoceramic-resin onlays (Grandio Blocs, VOCO GmbH, Germany)	Deep subgingival margins relocated supragingivally (DME) (four injectable materials)	Baseline, 1, 2, 3 years	Gingival Index GI; modified FDI criteria (fracture/retention, marginal adaptation, radiographic adaptation, hypersensitivity, vitality, recurrent caries, tooth integrity); survival	100% recall and 100% survival at 3 years. Significant increase in GI scores over time within groups, but no significant differences among materials at any evaluation period. No secondary caries, no loss of restorations, and no clinically unacceptable FDI scores. Injectable DME materials demonstrated comparable and acceptable clinical performance with only slight increase in gingival bleeding over 3 years.

All studies reported at least one of the prespecified outcomes, including restoration survival, marginal adaptation, secondary caries, and periodontal parameters such as bleeding on probing, plaque index, gingival index, and probing depth.

### 3.3 Descriptive analysis

The 10 included clinical studies were published between 2018 and 2026 across different countries, including Italy (19), the Netherlands (20), Egypt (10, 21), Turkey (22, 23, 25), Germany (5, 24), and Jordan (6). The study designs were primarily prospective clinical trials and controlled clinical studies (5, 19, 22–25), along with randomized controlled trials (10, 21), and a smaller number of retrospective analyses (6, 20). Sample sizes ranged from 20 teeth (21) to 197 teeth (20), with most studies enrolling between 20 and 80 posterior teeth. All studies focused on molars and premolars presenting with deep proximal subgingival margins, reflecting the clinical challenge of managing restorations in difficult isolation and bonding conditions.

In terms of intervention strategies, all studies evaluated DME using a variety of restorative materials. Composite-based materials were most commonly used, including flowable composites, bulk-fill resins, and highly filled injectable composites (5, 6, 19, 21–25). Some studies also investigated glass ionomer-based or bioactive materials as alternatives for margin elevation (10). Adhesive protocols varied, with several studies employing three-step etch-and-rinse systems (5, 20, 22–24), while others used universal adhesives in selective-etch or self-etch modes (6, 10, 19, 21, 25). Immediate dentin sealing was incorporated in selected protocols (10, 20). Luting procedures were predominantly performed using dual-cure resin cements or preheated composite resins to optimize bonding performance (5, 6, 20–25).

A wide range of indirect restorations was evaluated. Lithium disilicate restorations, including pressed and Computer-Aided Design/Computer-Aided Manufacturing (CAD/CAM)-fabricated partial crowns, were frequently reported (5, 19, 20, 24). Indirect resin composite restorations and CAD/CAM composite overlays were also commonly used (21–23), along with hybrid ceramic endocrown-like restorations (25) and zirconia- or nanoceramic-based restorations (6, 10). Comparators varied among studies and included conventional subgingival margins without relocation (5, 19, 24), supragingival or equigingival margins (5, 24, 25), and surgical crown lengthening procedures (21), highlighting different clinical approaches for managing deep margins.

Follow-up periods ranged from 6 months to 12 years. Most studies reported short- to medium-term outcomes between 1 and 3 years (5, 10, 21–25), while one retrospective study provided long-term data up to 12 years (20). Clinical evaluation criteria were comprehensive and included restoration survival and success rates (10, 20, 22–25), marginal adaptation and discoloration (10, 20, 22, 25), fracture incidence and retention loss (5, 20, 22–25), secondary caries (5, 10, 20, 22–25), and postoperative sensitivity (20, 22). Periodontal parameters were also extensively assessed, including bleeding on probing, plaque index, gingival index, probing pocket depth, clinical attachment level, and crestal bone level (5, 6, 10, 19, 21–25).

In terms of outcomes, DME techniques generally demonstrated high clinical success and survival rates, typically exceeding 93% across studies (22–25), with several reports indicating survival rates above 95% in short- to medium-term follow-up (10, 24, 25), and up to 12 years in long-term evaluation (20). Failures were limited and mainly associated with restoration fracture, debonding, secondary caries, or occasional endodontic complications (5, 20, 22–25).

Periodontal findings showed a consistent trend toward slightly increased gingival inflammation at DME sites, particularly reflected by higher bleeding on probing scores (5, 19, 24), especially when margins were located close to the bone crest (19). However, other periodontal parameters, including plaque accumulation, probing depth, and clinical attachment levels, remained stable and comparable to control groups over time (5, 6, 10, 21–25). Importantly, no significant radiographic bone loss or increased incidence of secondary caries was observed across the included studies (5, 10, 20–25).

Overall, despite variations in study design, materials, and restorative approaches, the included evidence consistently supports that DME is a reliable and minimally invasive technique for managing deep subgingival margins. The technique enables predictable adhesive procedures and favorable clinical outcomes, with survival rates comparable to conventional approaches and only minor, clinically manageable effects on periodontal health (5, 6, 10, 19–25).

### 3.4 Risk of bias of included studies

The methodological quality and risk of bias of the included clinical studies (5, 6, 10, 19–25) were evaluated using tools appropriate to study design. Randomized controlled trials were assessed using the Cochrane RoB 2 tool across five domains (D1–D5), while non-randomized and observational studies (prospective and retrospective) were evaluated using the ROBINS-I tool.

Based on the RoB 2 assessment, the included randomized controlled trials (10, 21) demonstrated overall low to moderate risk of bias. Roshdy et al. (10), was judged as having low risk of bias across all domains, reflecting appropriate randomization procedures, adequate allocation concealment, standardized outcome assessment, and complete outcome reporting. However, Farouk et al. (21), was judged as having some concerns, mainly related to deviations from intended interventions, as blinding of operators and participants was not feasible due to the nature of the clinical procedures. Nevertheless, both trials showed low risk in the domains of outcome measurement and selection of the reported results, supported by the use of standardized clinical evaluation criteria and predefined outcome measures. Overall, the methodological quality of the included randomized controlled trials was considered acceptable, with no study judged to be at high risk of bias.

The non-randomized studies, including prospective controlled clinical trials and retrospective analyses (5, 6, 19, 20, 22–25), were assessed using the ROBINS-I tool. Overall, most studies were judged to have a moderate risk of bias, while one retrospective study (6) was rated as having a serious risk of bias, resulting in an overall body of evidence with predominantly moderate methodological quality. The primary sources of bias were related to confounding (Domain 1) and selection of participants (Domain 2). In several studies, the absence of randomization and allocation based on clinical indications (e.g., margin depth or defect severity) introduced potential baseline imbalances. Although prospective designs and, in some cases, within-subject comparisons partially mitigated confounding, residual bias could not be excluded. Retrospective designs, particularly that of El-Ma'aita et al. (6), were more susceptible to serious confounding and selection bias due to lack of control over baseline characteristics and patient inclusion. Bias in the classification of interventions (Domain 3) was consistently judged as low across all studies, as interventions such as DME were clearly defined and clinically distinguishable. Similarly, bias due to deviations from intended interventions (Domain 4) was generally low, reflecting well-described and standardized clinical protocols across studies. Regarding missing data (Domain 5), most studies demonstrated low to moderate risk. While several prospective trials reported complete or near-complete follow-up, others exhibited partial attrition over time [e.g., Hausdörfer et al. (24)], which may have introduced bias where not fully addressed. However, this was not sufficient to elevate most studies beyond moderate risk. Bias in the measurement of outcomes (Domain 6) was predominantly low, supported by the use of validated and widely accepted clinical indices, including United States Public Health Service (USPHS), Fédération Dentaire Internationale (FDI) criteria, and periodontal parameters (bleeding on probing, plaque index, probing pocket depth). In multiple studies, outcome assessors were blinded and calibrated, further strengthening measurement reliability. Finally, bias in the selection of the reported result (Domain 7) was judged as low across the included studies, as outcomes were generally consistent with study objectives and, in some cases, supported by trial registration and predefined protocols.

**Figure 2:**
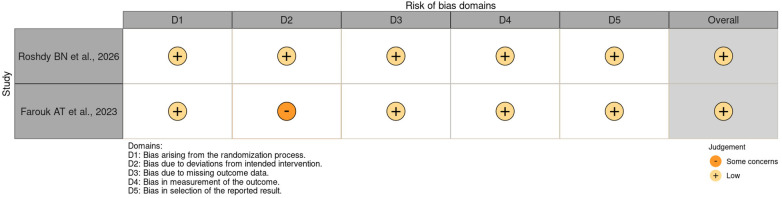
Summary of risk of bias assessment of randomized controlled studies using the cochrane risk of bias 2 (RoB 2) tool.

**Figure 3:**
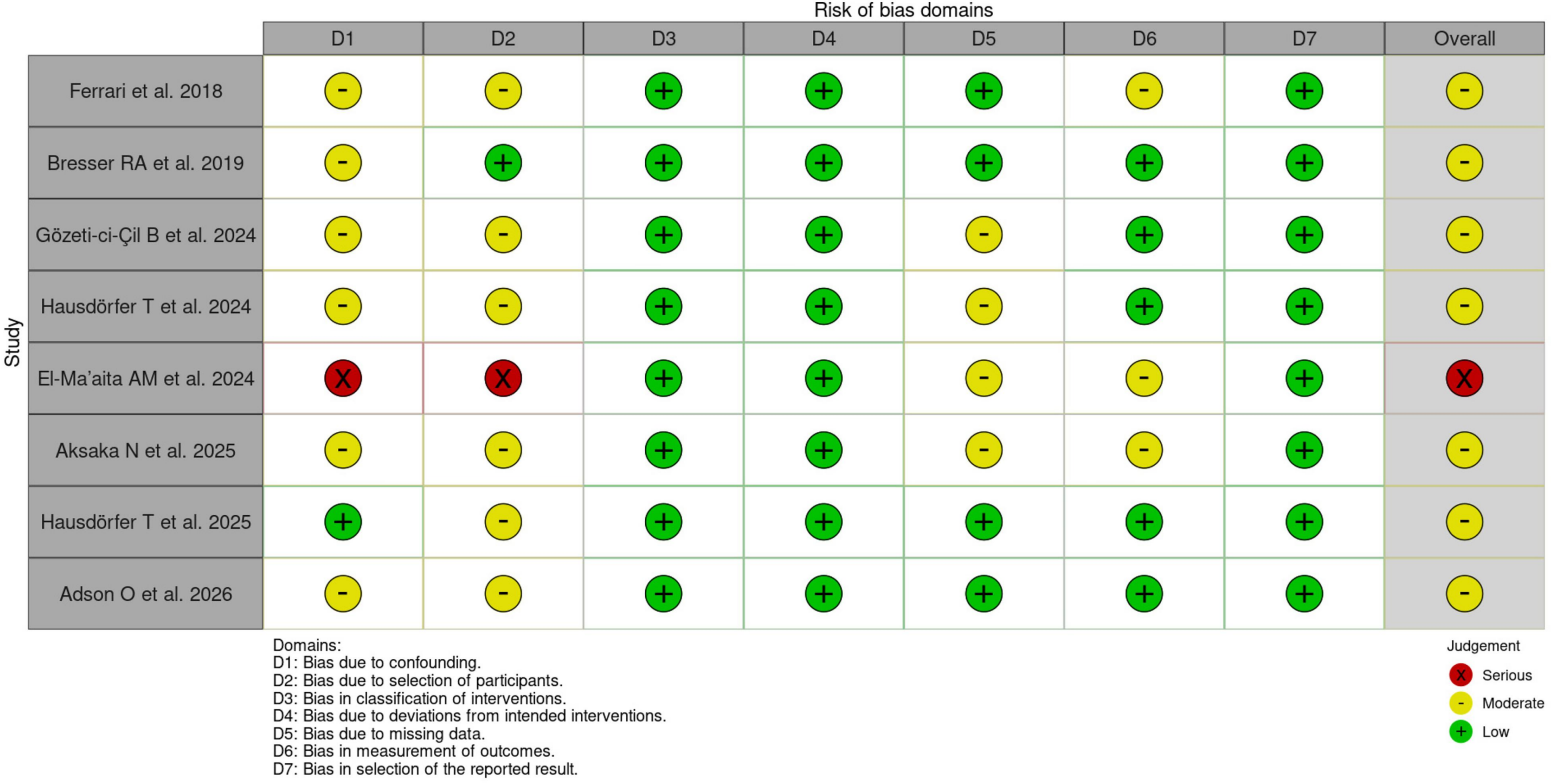
Summary of risk of bias assessment of non-randomized studies using the risk of bias in Non-randomized studies—of interventions (ROBINS-I) tool.

A detailed summary of the domain-specific risk of bias assessments is presented in Figures 2, 3. Overall, while the included evidence demonstrates acceptable methodological quality, the presence of moderate risk in most studies and serious risk in one retrospective study should be carefully considered when interpreting the findings.

### 3.5 Evidence quality

The certainty of evidence for clinical outcomes of DME was evaluated using the GRADE approach (Table 3). Evidence derived from the two randomized controlled trials (10, 21) provides moderate-certainty evidence that DME using composite-based materials (flowable, bulk-fill, and injectable composites) yields comparable short- to medium-term restoration survival and marginal adaptation relative to conventional approaches (supragingival or equigingival margins, or surgical crown lengthening). Downgrading was primarily due to some concerns regarding risk of bias, including the impossibility of operator blinding and small sample sizes, as well as imprecision related to limited clinical events over the follow-up periods.

**Table 3:** Grading of recommendations, assessment, development and evaluation (GRADE) summary of evidence for clinical outcomes of deep margin elevation (DME) technique.

Clinical Outcome	No. of Studies (Teeth)	Study Design	Risk of Bias	Inconsistency	Indirectness	Imprecision	Publication Bias	Overall Certainty of Evidence (GRADE)
Restoration survival/success (1–12 years)	7 studies (∼623 teeth)	RCTs, prospective and retrospective clinical studies	Seriousᵃ	Not serious	Not serious	Seriousᵇ	Undetected	⊕⊕⊕⊝ Moderate
Marginal adaptation/restoration integrity (FDI/USPHS criteria)	5 studies (∼469 teeth)	Prospective and retrospective clinical studies	Seriousᵃ	Not serious	Not serious	Seriousᵇ	Undetected	⊕⊕⊕⊝ Moderate
Secondary caries incidence	7 studies (∼566 teeth)	Clinical studies	Seriousᵃ	Not serious	Not serious	Very seriousᶜ	Undetected	⊕⊕⊝⊝ Low
Postoperative sensitivity/hypersensitivity	3 studies (∼361 teeth)	Clinical studies	Seriousᵃ	Not serious	Possible seriousᵈ	Seriousᵇ	Undetected	⊕⊕⊝⊝ Low
Periodontal outcomes (BOP, PI, GI, PPD, CAL, bone level)	8 studies (∼459 teeth)	RCTs and prospective clinical studies	Seriousᵃ	Not serious	Not serious	Seriousᵇ	Undetected	⊕⊕⊕⊝ Moderate
Restoration fracture/debonding/technical complications	5 studies (∼518 teeth)	Clinical studies	Seriousᵃ	Not serious	Not serious	Seriousᵇ	Undetected	⊕⊕⊕⊝ Moderate

a Serious risk of bias: predominance of non-randomized clinical designs, heterogeneous restorative materials and margin depths, limited blinding of outcome assessment.

b Serious imprecision: relatively small sample sizes per outcome group, limited number of failure events, and variability in follow-up duration.

c Very serious imprecision: secondary caries occurred rarely across studies, resulting in wide uncertainty in effect estimates.

d Possible serious indirectness: postoperative sensitivity measured using heterogeneous clinical or patient-reported assessment protocols and varying timepoints.

For secondary outcomes, including secondary caries, postoperative sensitivity, and periodontal parameters (bleeding on probing, probing depth, clinical attachment level), the certainty of evidence was low, primarily due to serious imprecision (limited number of events and modest sample sizes), potential indirectness (variability in outcome definitions, follow-up durations, and assessment methods), and risk of bias in one of the randomized controlled trials (21). While the available data suggest no clinically meaningful adverse effects associated with DME, confidence in these estimates is limited, and results should be interpreted cautiously.

Evidence from the non-randomized studies [prospective controlled trials and retrospective analyses (5, 6, 19, 20, 22–25)] was judged as low to very low certainty. Although these studies consistently reported high survival rates (>93%) and minor, clinically manageable periodontal effects, the presence of moderate to serious risk of bias (primarily due to confounding and participant selection), heterogeneity in restorative materials and adhesive strategies, and limited follow-up data reduced confidence in the estimates. These findings should be considered supportive or hypothesis-generating rather than confirmatory.

Overall, the body of evidence suggests that DME is a reliable and minimally invasive technique for managing deep subgingival margins in posterior teeth, enabling predictable adhesive procedures and favorable clinical outcomes. However, moderate risk of bias in randomized controlled trials and predominantly low-certainty evidence from non-randomized studies warrant cautious interpretation, particularly regarding long-term effects on periodontal health and rare complications.

### 3.6 Meta-analysis

A meta-analysis of the included clinical studies (5, 6, 10, 19–25) was conducted, with separate analyses for fracture or debonding, secondary caries, and gingival inflammation/bleeding outcomes following indirect restorations with or without DME.

**Figure 4:**
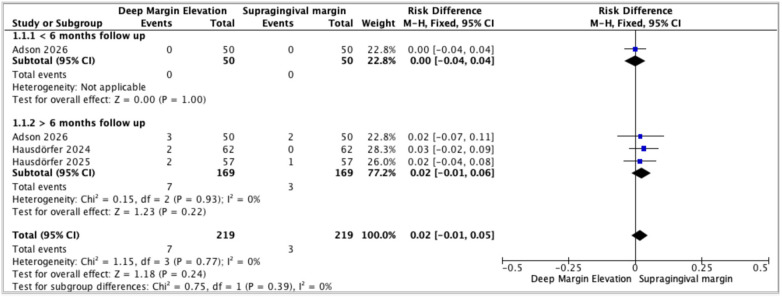
Forest plot comparing fracture or debonding outcomes between DME and control groups.

For fracture or debonding of restorations (Figure 4), no statistically significant difference was observed between restorations placed with DME and control groups. The overall pooled analysis demonstrated comparable performance between groups (*p* = 0.77). Subgroup analyses based on follow-up duration (≤6 months and >6 months) also revealed no statistically significant differences, indicating consistent mechanical stability of restorations regardless of margin elevation.

**Figure 5:**
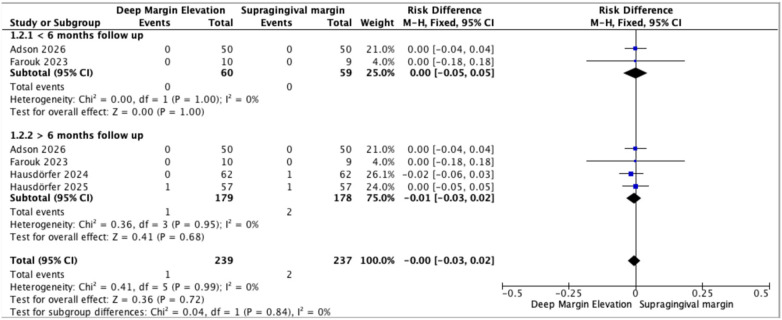
Forest plot comparing secondary caries outcomes between DME and control groups.

Similarly, the meta-analysis of secondary caries (Figure 5) demonstrated no statistically significant differences between groups. At short-term follow-up (<6 months), no difference was detected (*p* = 1.00), and this finding remained consistent at longer follow-up periods (>6 months) (*p* = 0.95). The overall pooled analysis confirmed equivalent incidence of secondary caries between DME and non-DME restorations, suggesting that margin elevation does not adversely affect caries risk.

**Figure 6:**
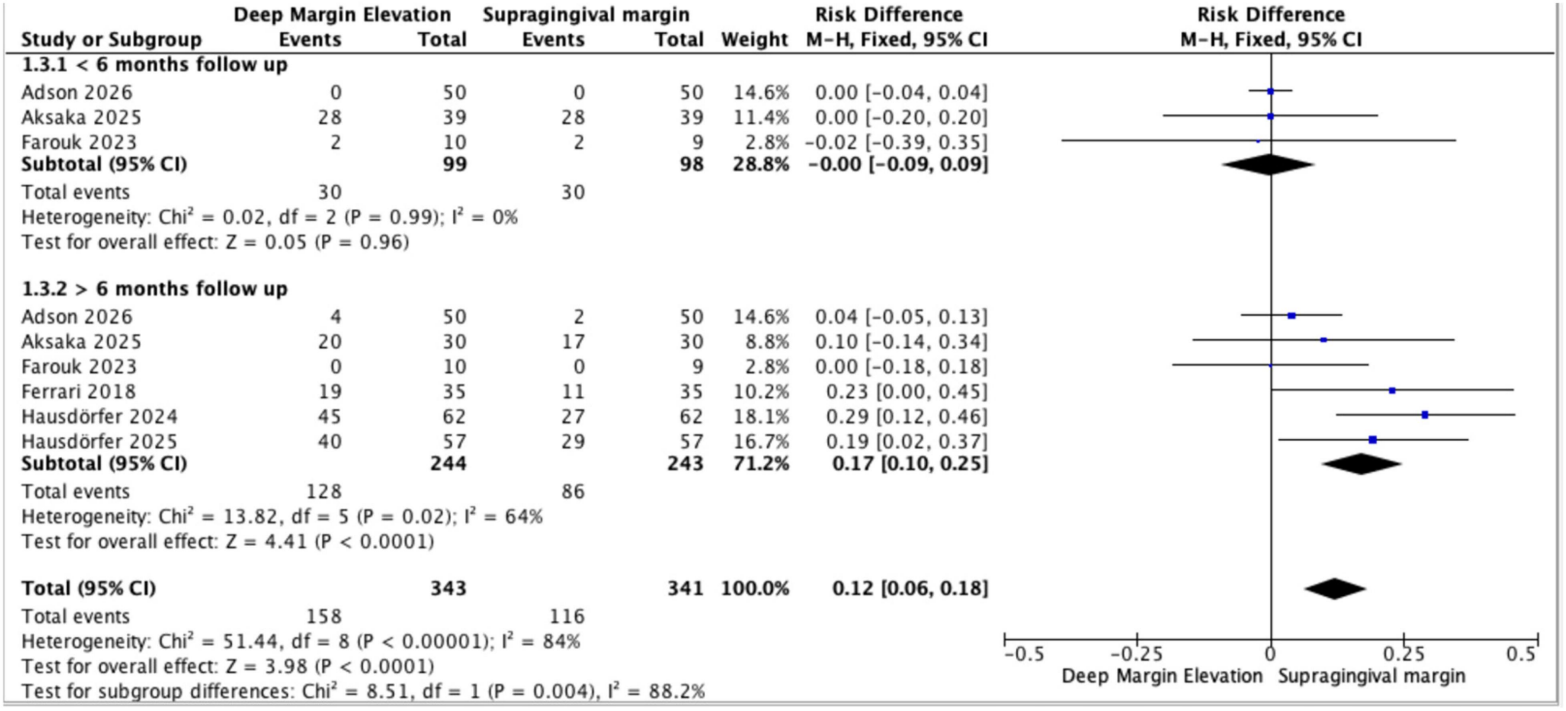
Forest plot comparing gingival inflammation and bleeding outcomes between DME and control groups.

In contrast, the analysis of gingival inflammation and bleeding (Figure 6) revealed time-dependent differences. At short-term follow-up (≤6 months), no statistically significant difference was observed between groups (*p* = 0.99). However, at longer follow-up periods (>6 months), restorations placed after DME exhibited a statistically significant increase in gingival inflammation and bleeding compared with controls (*p* = 0.02). The overall pooled analysis further confirmed this finding (*p* < 0.001). Importantly, the absolute increase in bleeding on probing across studies ranged from 4% to 12%, with mean probing pocket depths remaining below 4 mm in all groups. No study reported a statistically or clinically significant increase in clinical attachment loss or radiographic bone resorption associated with DME. Thus, while statistically significant, the observed difference may not represent clinically meaningful periodontal compromise when DME is performed within biological limits.

Overall, the meta-analysis indicates that DME does not negatively influence restoration integrity or secondary caries occurrence but may be associated with a modest increase in gingival inflammation over time, particularly beyond 6 months of clinical service.

**Figure 7:**
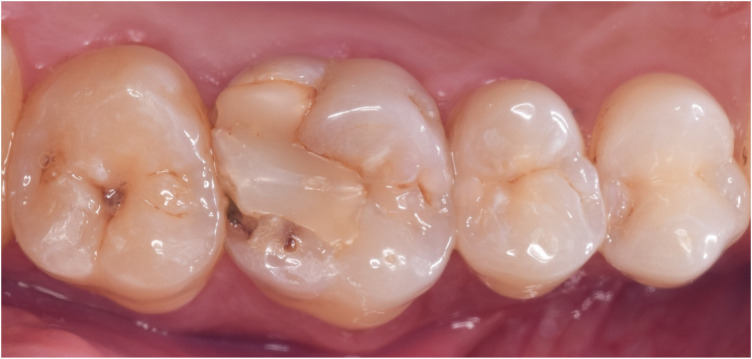
A preoperative situation shows recurrent caries around old composite restoration in the distal half of the upper first molar and two proximal carious lesions in the mesial side of the upper first molar and the distal side of the second premolar.

**Figure 8:**
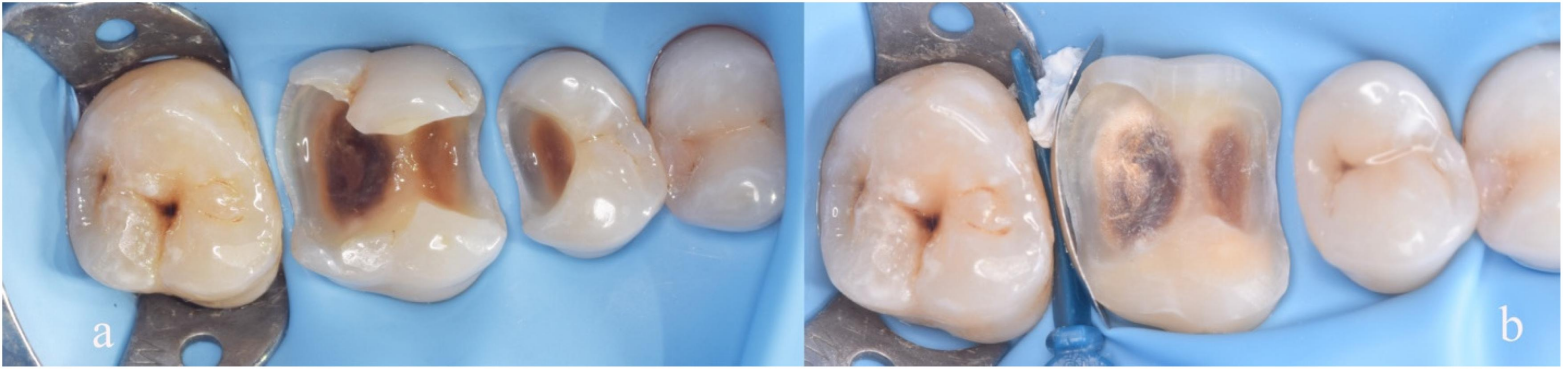
**(a)** Selective caries removal obtaining a peripheral seal zone for both teeth with a decision to protect the structurally compromised first molar with an indirect overlay, **(b)** overaly preparation and matricing to perform DME and cavity design optimization for the first molar and a direct restoration was completed for the second premolar.

**Figure 9:**
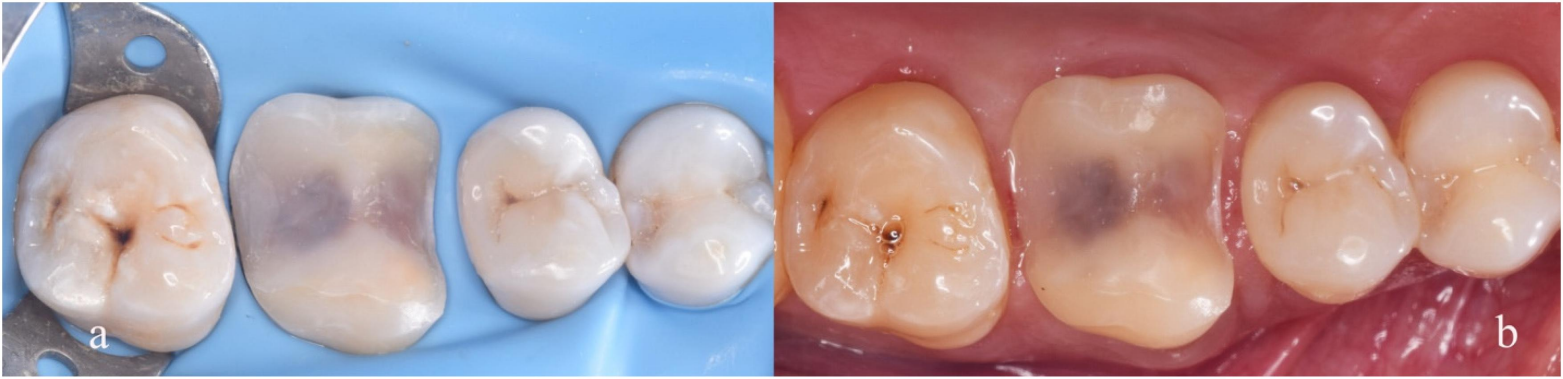
**(a)** Foundation restoration was completed, **(b)** final preparation with refined enamel margins ready for impression recording.

**Figure 10:**
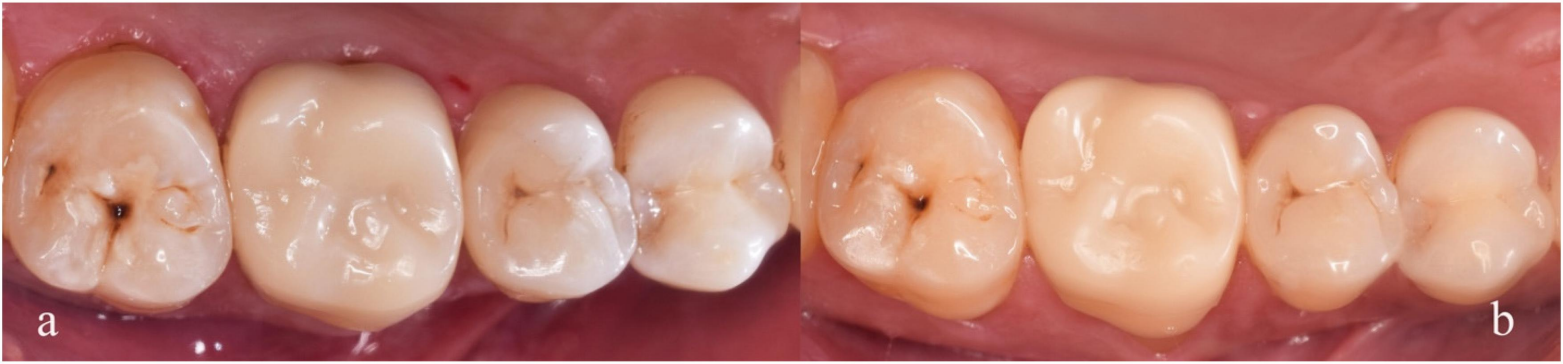
**(a)** Immediate postoperative after cementation of indirect composite restoration, **(b)** 11 months follow-up shows the periodontal stability around the indirect restoration and a completely integrated direct restoration in the second premolar.

To illustrate the clinical applicability of these findings, a representative case is presented (Figures 7–10). A preoperative examination revealed recurrent caries associated with an existing composite restoration on the distal aspect of the maxillary first molar, along with proximal carious lesions involving the mesial surface of the same tooth and the distal surface of the adjacent second premolar. Following selective caries removal, a peripheral seal zone was established, and due to structural compromise, the first molar was restored using an indirect overlay restoration. DME was performed to relocate the cervical margin to a more accessible supragingival position, facilitating adhesive procedures and isolation. In contrast, the second premolar, presenting with less extensive tissue loss, was managed with a direct restoration. A foundation restoration was subsequently completed, followed by final tooth preparation with refined enamel margins. After adhesive cementation of the indirect restoration, the clinical outcome was evaluated at 11 months, demonstrating satisfactory periodontal conditions, absence of complications, and optimal integration of both the indirect overlay and the direct restoration. When an appropriate clinical protocol is strictly followed, the potential increase in gingival inflammation over time, particularly beyond 6 months, may be minimized and may not be clinically significant.

## 4 Discussion

The present systematic review and meta-analysis aimed to evaluate whether the type of material used for DME influences the clinical performance of indirect restorations. Based on the available clinical evidence, no statistically significant differences were observed among different DME materials with respect to restoration survival (fracture or debonding) and secondary caries. However, a statistically significant increase in gingival inflammation and bleeding on probing was observed at DME sites over time. Therefore, the null hypothesis is partially rejected, as differences were identified in periodontal outcomes, while no significant differences were found for the remaining evaluated clinical parameters.

One of the most relevant findings of this review is the consistently high survival rate of restorations placed following DME, ranging from 93% to 100% over follow-up periods extending up to 12 years (5, 10, 20, 22–25). These results are comparable to survival rates reported for restorations placed on supragingival or equigingival margins, supporting the concept that DME does not compromise the longevity of indirect restorations (24, 25). The meta-analysis further confirmed that there were no statistically significant differences in fracture or debonding rates between DME and control groups (*p* = 0.77), indicating that the mechanical integrity of restorations is preserved following margin relocation. These findings are consistent with previous clinical and laboratory studies demonstrating that adequate adhesive protocols and proper stress distribution can compensate for the presence of deep margins (11, 13, 24, 25). Another plausible explanation for this finding lies in the fact that failure in adhesive restorations is predominantly governed by the quality of the tooth-cement interface rather than the vertical position of the restorative margin. Clinical observations have shown that, in cases of debonding, failure mainly occurs at the tooth-cement interface, while the luting material remains adherent to the restoration, indicating that bonding to Computer-Aided Design/Computer-Aided Manufacturing (CAD/CAM) materials is generally reliable (26, 27). Therefore, the presence of DME does not introduce a new weak interface but rather relocates the margin coronally, often facilitating improved access, visibility, and adhesive control (24, 25).

In addition, contemporary adhesive systems and resin-based luting protocols are capable of compensating for the challenges associated with subgingival margins by providing adequate micromechanical and chemical bonding to dentin (11–13). When proper isolation techniques, such as rubber dam application, and optimized adhesive strategies are employed, the integrity of the bonded interface can be maintained irrespective of margin depth (28–31). This may explain why no significant differences in fracture or debonding were observed between groups.

Furthermore, the limited number of mechanical failures reported across studies suggests that such events are relatively rare and may be more strongly influenced by factors such as occlusal loading, cavity design, and remaining tooth structure rather than the use of DME itself (20, 32). The consistency of these findings with previous clinical studies reporting comparable survival rates for restorations with and without DME further supports the concept that DME does not compromise mechanical stability (24, 25).

Secondary caries and postoperative sensitivity were infrequently reported across the included studies and did not differ significantly between DME and control groups (*p* > 0.05) (23–25). A plausible explanation for this finding is that the occurrence of secondary caries is multifactorial and not solely dependent on the presence of margin elevation. Clinical evidence suggests that patient-related factors, particularly individual caries risk, play a decisive role in the development of secondary caries. In several studies, the exclusion of high caries risk patients may have contributed to the absence or low incidence of caries in both groups, thereby limiting the ability to detect potential differences (23–25).

In addition, marginal integrity remains a critical determinant for secondary caries formation (33). Although DME involves the creation of an additional interface, the use of contemporary adhesive systems and meticulous clinical execution may ensure adequate marginal sealing, thereby minimizing bacterial infiltration (1, 2, 7, 24). Conversely, it has been suggested that the oral environment, particularly saliva contamination, may have a greater influence on caries development than the restorative technique itself (34). In this context, the consistent use of rubber dam isolation in many of the included studies likely played a key role in reducing contamination and improving adhesive performance, which may explain the absence of secondary caries in both DME and control groups (23–25, 35). This is supported by evidence showing that restorations performed under rubber dam isolation exhibit lower failure rates compared to those placed using cotton roll isolation alone (28–31).

The management of deep subgingival margins presents significant technical challenges, particularly in terms of matrix adaptation and moisture control. Achieving proper matrix sealing in these conditions is essential to ensure adequate marginal integrity and to prevent overhangs or gaps that may compromise periodontal health. However, the proximity to gingival tissues and the presence of crevicular fluid often make isolation difficult, increasing the risk of contamination during adhesive procedures. In this context, the rubber dam isolation plays a critical role in improving the clinical success of DME by providing a controlled and dry working field. Additionally, Immediate Dentin Sealing (IDS), when applied prior to restoration placement, has been shown to enhance bond strength, reduce microleakage, and improve the durability of adhesive interfaces (36). Therefore, the variability in the application of matrixing techniques, isolation methods, and IDS protocols across the included studies may partially explain differences in clinical outcomes. These factors should be carefully standardized in future research and considered essential components of clinical protocols when performing DME.

Furthermore, the relatively short follow-up periods reported in several studies may have limited the detection of late biological complications, including secondary caries (5, 10, 21–25). It is well established that caries recurrence is a time-dependent process, and longer observation periods are required to accurately assess its incidence. Additionally, technical factors such as the presence of voids or defects at the restoration interface may contribute to caries development, emphasizing the importance of careful restorative technique regardless of the use of DME (37).

Therefore, the absence of significant differences between groups should not be interpreted as definitive evidence of equivalence but rather as a reflection of controlled clinical conditions, appropriate case selection, and optimized adhesive protocols. This interpretation is further supported by the low certainty of evidence for these outcomes, as indicated by the GRADE assessment, highlighting the need for cautious interpretation and well-designed long-term clinical studies.

In contrast to mechanical and biological outcomes, periodontal evaluation revealed a statistically significant increase in gingival inflammation and bleeding on probing at DME sites beyond 6 months (*p* = 0.02), as confirmed by the meta-analysis. However, a distinction must be made between statistical and clinical significance. The pooled absolute increase in bleeding on probing across studies ranged from 4% to 12%, with mean BoP values at DME sites ranging from 12% to 24% compared with 8% to 16% at control sites. Several included studies have reported localized inflammatory responses at relocated margins, particularly when margins were positioned close to the alveolar bone crest (5, 19, 24). Despite this statistically significant difference, the clinical relevance is questionable for two reasons. First, probing depths generally remained stable and below 4 mm across all studies, with mean values ranging from 2.6 mm to 3.2 mm. Second, clinical attachment levels and radiographic bone heights were not significantly affected, indicating that the inflammatory response was confined to the gingival tissues without progression to periodontitis (24, 38, 39). According to the 2017 World Workshop classification, isolated bleeding on probing without attachment loss or bone resorption is classified as gingival inflammation (health on a reduced periodontium) rather than periodontitis (40, 41). Therefore, while statistically significant, the observed increase in bleeding on probing is clinically modest and manageable with appropriate oral hygiene measures.

Importantly, although bleeding on probing was slightly increased at DME sites, probing depths generally remained stable and below 3 mm, while clinical attachment levels and radiographic bone heights were not significantly affected (24, 38, 39). This suggests that the inflammatory changes were mild, clinically manageable, and did not compromise periodontal health over the evaluated follow-up periods. Consistent with previous studies, the increase in bleeding on probing appeared to stabilize over time, with no further progression between 1- and 2-year follow-ups, indicating that the initial inflammatory response is transient and largely controlled by patient oral hygiene and supportive care (40, 41). The gingival index, used in several studies as a key indicator of periodontal health, reinforces the importance of monitoring inflammatory responses at DME sites (42).

The observed differences between DME and control sites may also reflect technical factors, such as marginal adaptation, cement removal, and operator experience, rather than a fundamental biological disadvantage of margin elevation (24, 25, 27, 28). Moreover, the use of biocompatible, highly filled flowable composites and adherence to isolation protocols (e.g., rubber dam application) likely contributed to the preservation of periodontal stability (24, 25, 43). Consistent plaque index values between DME and control groups further support the conclusion that biofilm accumulation is comparable, and that minor increases in gingival inflammation are not necessarily associated with clinical disease progression (4, 18).

The observed increase in bleeding on probing and localized gingival inflammation at DME sites may be closely related to the proximity of restoration margins to the alveolar bone crest. When restorative margins are placed too close to the crestal bone, there is a potential risk of violating the supracrestal tissue attachment, which plays a critical role in maintaining periodontal health. This biological disruption may lead to a persistent inflammatory response, clinically expressed as bleeding on probing and gingival irritation. Such findings suggest that, although DME can be a valuable technique for managing subgingival margins, careful consideration must be given to the vertical positioning of the restorative margin. From a clinical perspective, maintaining an adequate distance between the restoration margin and the alveolar crest is essential to preserve periodontal stability and minimize the risk of long-term complications. These results highlight the importance of proper case selection, precise margin elevation, and adherence to biological principles when performing DME procedures.

Overall, these findings indicate that, while DME may induce a modest transient increase in bleeding on probing at relocated margins, periodontal health remains clinically stable when the procedure is performed correctly, and inflammation does not progress to attachment loss or radiographic bone resorption. This emphasizes the importance of case selection, careful restorative technique, respect for the biological width, and supportive oral hygiene protocols when performing DME (4, 6, 10, 24, 25, 27, 28).

Regarding material selection, composite-based materials, including flowable, bulk-fill, and injectable composites, demonstrated comparable clinical performance across all primary outcomes (5, 6, 19, 21–25). This finding is noteworthy given theoretical differences in polymerization shrinkage stress and elastic modulus mismatch that have been shown *in vitro* to influence interfacial integrity (1, 11, 12). Flowable composites typically exhibit higher shrinkage but lower modulus, potentially allowing stress dissipation through material deformation, whereas bulk-fill composites incorporate modified resin matrices specifically designed to reduce polymerization stress (20). DME creates a layered interface (elevation material–luting cement–indirect restoration), and modulus mismatch between layers could theoretically concentrate stress at the bonded interfaces (24). However, the comparable fracture and debonding rates observed between DME and control groups suggest that these theoretical concerns are clinically manageable, likely due to the buffering effects of dual-cure luting cements (which polymerize slowly and generate lower shrinkage stress) and the use of rubber dam isolation, which eliminates moisture contamination as a confounding variable (11).

Adhesive strategy represents a critical confounding variable that has received insufficient attention. Across the included studies, protocols varied substantially, including three-step etch-and-rinse systems (OptiBond FL), universal adhesives applied in selective-etch or self-etch modes, and immediate dentin sealing (5, 6, 10, 20–25). These strategies differ markedly in their ability to achieve durable bonding to dentin (20). The consistent clinical outcomes across different elevation materials, despite this adhesive protocol heterogeneity, suggests that achieving a stable, well-sealed dentin interface may be more critical than the specific elevation material—a finding consistent with evidence that failure in adhesive restorations typically occurs at the tooth-cement interface rather than within the bulk material (26, 27).

In addition, the use of resin-modified glass ionomer cements (RMGICs) as DME materials has been proposed due to their chemical adhesion to dentin and fluoride-releasing properties (10). While acceptable outcomes were reported with these materials, concerns remain regarding their inferior mechanical properties and long-term stability under indirect restorations compared with resin composites (10). Nevertheless, the limited number of clinical studies evaluating RMGICs precludes definitive conclusions, and further research is needed to determine their suitability in DME procedures.

These findings underscore the importance of adhering to fundamental periodontal principles when performing DME. Specifically, maintaining an adequate distance between the restoration margin and the alveolar crest, ensuring smooth and well-polished surfaces, and implementing strict plaque control measures are essential to minimize periodontal complications (2, 19). In this context, DME should not be considered a universal alternative to surgical crown lengthening but rather a technique that must be applied within biologically acceptable limits. When these limits are exceeded, surgical or orthodontic approaches may still be indicated to ensure long-term periodontal stability (3).

Another important aspect to consider is the heterogeneity among the included studies. The included studies exhibited clinical diversity in study design, comparator margin location (supragingival, equigingival, unprepared subgingival, or surgical crown lengthening), and adhesive protocols (three-step etch-and-rinse, universal adhesives, immediate dentin sealing). These variations represent potential threats to the validity of pooled effect estimates. The heterogeneity observed means that the pooled estimate for gingival inflammation-a statistically significant increase in bleeding on probing-should be interpreted with greater caution, as the true effect may vary across clinical scenarios. The absence of uniform comparator definitions and the inability to perform subgroup analyses due to the limited number of studies per category further constrain the precision of our conclusions. These limitations are inherent to systematic reviews of emerging techniques and underscore the need for standardized reporting in future DME research. These limitations are reflected in the GRADE assessment, which rated the certainty of evidence as moderate for primary outcomes and low for several secondary outcomes. Furthermore, the present review exclusively evaluated DME in combination with indirect restorations, as this reflected the available clinical evidence meeting our inclusion criteria. The use of DME with direct composite restorations, while clinically common, remains under-investigated in the literature. Therefore, the findings of this review should not be extrapolated to DME performed prior to direct restorations without further evidence.

Furthermore, most studies included in this review reported short- to medium-term outcomes, with only one study providing long-term data up to 12 years (20). Although the available evidence suggests that DME is a reliable technique, the long-term biological and mechanical behavior of relocated margins remains insufficiently documented. Future studies should aim to provide long-term data with standardized protocols and clearly defined outcome measures to strengthen the evidence base.

From a clinical perspective, the findings of this review support the integration of DME into contemporary adhesive dentistry as part of a minimally invasive and biomimetic treatment approach. The technique allows clinicians to preserve tooth structure, avoid invasive surgical procedures, and optimize adhesive conditions for indirect restorations (2, 4). However, the success of DME is highly technique-sensitive and depends on proper case selection, meticulous execution, and strict adherence to adhesive and periodontal principles.

The heterogeneity observed among the included studies should be carefully considered when interpreting the results. The wide variation in follow-up durations, ranging from short-term evaluations to long-term assessments, may lead to an underestimation of late complications such as secondary caries or periodontal breakdown in studies with limited observation periods. In addition, the inclusion of different restorative materials, particularly light-activated resin composites and resin-modified glass ionomer cements, introduces variability in mechanical properties, bonding behavior, and clinical performance. This heterogeneity limits direct comparability across studies and may contribute to inconsistencies in reported outcomes. Therefore, caution should be exercised when extrapolating these findings to clinical practice, and future studies with standardized protocols and longer follow-up periods are recommended.

Another important limitation of the current evidence base is the predominance of studies evaluating resin composite materials, particularly flowable, bulk-fill, and injectable composites. Although this review aimed to assess a broader range of polymer-based materials, including resin-modified glass ionomer cements and bioactive alternatives, data on these materials remain scarce. This imbalance limits the generalizability of the findings and restricts the ability to perform meaningful comparisons across different material classes. As a result, the conclusions drawn from this review are primarily applicable to composite-based restorative approaches. Future research should focus on expanding the evidence base for alternative polymer-based materials, particularly those with potential bioactive or ion-releasing properties, to better understand their clinical performance in deep margin elevation procedures.

Also, further investigations should focus on well-designed randomized controlled trials with larger sample sizes and standardized methodologies. In particular, direct comparisons between different composite formulations under controlled clinical conditions are needed to determine whether specific material properties may offer advantages in certain clinical scenarios. Key outcomes should include periodontal parameters (bleeding on probing, probing depth, clinical attachment level), restoration survival, marginal adaptation, secondary caries, and failure patterns. Such studies would substantially enhance the clinical applicability of the DME evidence base. Further investigation into the interaction between DME and periodontal tissues is essential to better understand the biological implications of margin relocation and to refine clinical guidelines. Emerging materials in the dental market, such as the Stela Self-Cure Bulk-Fill Composite (SDI, Victoria, Australia), could be implemented in future studies to evaluate their performance in DME protocols and assess potential benefits in terms of handling, mechanical properties, and biological compatibility.

## 5 Conclusions

Within the limitations of the available clinical evidence, DME appears to be a reliable and minimally invasive technique for managing subgingival proximal margins in teeth restored with indirect restorations. Composite-based polymeric materials, including flowable, bulk-fill, and injectable resins, demonstrate comparable clinical performance, with no clear superiority identified among them.

DME does not adversely affect restoration survival, mechanical stability, or the incidence of secondary caries. However, a statistically significant but clinically modest increase in gingival inflammation and bleeding on probing may occur over time, particularly when margins are positioned close to the periodontal attachment. While clinicians should be aware of this finding, it should not discourage the use of DME when indicated, given that appropriate case selection, meticulous restorative technique, and regular periodontal maintenance are ensured.

Therefore, successful implementation of DME depends not only on material selection but also on strict adherence to biological principles, precise margin placement, and meticulous clinical execution. Further high-quality randomized controlled trials with long-term follow-up are required to strengthen the evidence base and establish definitive clinical guidelines for material selection in DME procedures.

## Data Availability

The original contributions presented in the study are included in the article/Supplementary Material, further inquiries can be directed to the corresponding author.
